# Curbing Alcohol Use in Male Adults Through Computer Generated Personalized Advice: Randomized Controlled Trial

**DOI:** 10.2196/jmir.1695

**Published:** 2011-06-30

**Authors:** Brigitte Boon, Anneke Risselada, Annemarie Huiberts, Heleen Riper, Filip Smit

**Affiliations:** ^7^VU University Medical CentreDepartment of Epidemiology and BiostatisticsAmsterdamNetherlands; ^6^VU UniversityDepartment of Clinical PsychologyAmsterdamNetherlands; ^5^Trimbos-instituteInnovation Center for Mental Health and TechnologyUtrechtNetherlands; ^4^Netherlands Institute for Health Promotion and Disease PreventionWoerdenNetherlands; ^3^Municipal Health AuthoritySouth-Holland South regionNetherlands; ^2^Addiction Research InstituteRotterdamNetherlands; ^1^Trimbos-instituteDepartment of Public Mental HealthUtrechtNetherlands

**Keywords:** Web-based personalized feedback, alcohol, Internet, heavy drinking, problem drinking, adult

## Abstract

**Background:**

In recent years, interventions that deliver online personalized feedback on alcohol use have been developed and appear to be a feasible way to curb heavy drinking. Randomized controlled trials (RCTs) among the general adult population, however, are scarce. The present study offers an RCT of Drinktest.nl, an online personalized feedback intervention in the Netherlands.

**Objective:**

The aim of this study was to assess the effectiveness of computer-based personalized feedback on heavy alcohol use in male adults.

**Methods:**

Randomization stratified by age and educational level was used to assign participants to either the intervention consisting of online personalized feedback or an information-only control condition. Participants were told as a cover story that they would evaluate newly developed health education materials. Participants were males (n = 450), aged 18 to 65 years, presenting with either heavy alcohol use (> 20 units of alcohol weekly) and/or binge drinking (> 5 units of alcohol at a single occasion at least 1 day per week) in the past 6 months. They were selected with a screener from a sampling frame of 25,000 households. The primary outcome measure was the percentage of the participants that had successfully reduced their drinking levels to below the Dutch guideline threshold for at-risk drinking.

**Results:**

Intention-to-treat analysis showed that in the experimental condition, 42% (97/230) of the participants were successful in reducing their drinking levels to below the threshold at the 1-month follow-up as compared with 31% (67/220) in the control group (odds ratio [OR] = 1.7, number needed to treat [NNT] = 8.6), which was statistically significant (χ^2^_1_ = 6.67, *P* = .01). At the 6-month follow-up, the success rates were 46% (105/230) and 37% (82/220) in the experimental and control conditions, respectively (OR = 1.4, NNT = 11.9), but no longer statistically significant (χ^2^_1_ = 3.25, *P* = .07).

**Conclusions:**

Personalized online feedback on alcohol consumption appears to be an effective and easy way to change unhealthy drinking patterns in adult men, at least in the short-term.

**Trial registration:**

International Standard Randomized Controlled Trial Number: NTR836; http://www.trialregister.nl/trialreg/admin/rctview.asp?TC=836 (Archived by WebCite at http://www.webcitation.org/5ytnEz2vp)

## Introduction

The present study aims to examine the effect of Drinktest (www.drinktest.nl), an online personalized feedback intervention targeted at heavy drinking adults in the Netherlands. It is important to inform heavy drinkers about the possible consequences of their drinking behavior. After all, heavy alcohol consumption is highly prevalent worldwide [1] and is associated with a significant disease burden [2] and a range of health-related adverse consequences in later life, such as liver cirrhosis, cancer of the esophagus and the stomach, and possibly the onset of depressive disorder [3]. Heavy drinking is not only associated with morbidity, but also with excess mortality [4]. For these reasons alone, there is wisdom in reducing heavy alcohol use. This is likely to also reduce the risk of a host of other problems, such as crime, domestic violence, and traffic accidents. In addition, it may result in cost savings not only in health care (eg, fewer hospital stays), but it may also be advantageous for the economy when people are less often absent from their work and are more efficient while at work [3,5].

Unfortunately, it is not easy to reach heavy drinkers with face-to-face interventions. There often is a shortage of health care professionals who can deliver the interventions, even in resource rich countries. Moreover, heavy drinkers may be reluctant to discuss their drinking behavior [6]. As a consequence, a substantial 80% of heavy drinkers do not engage in any formal treatment [7].

Offering interventions online may help to solve this problem. Online interventions designed to decrease alcohol consumption have proven to be feasible instruments to reach heavy drinkers and are generally well received [8,9]. People can engage in the intervention whenever they choose and in the privacy of their home without fear of stigmatization. Moreover, online self-help interventions require no therapist time. Evidence regarding the effectiveness of online interventions is slowly building up. Results are promising [8,10].

Most of the evidence regarding the effectiveness of online alcohol interventions is collected in studies aimed at college students. In a recent meta-analysis, Carey and colleagues [11] found that computer-delivered interventions produced significant improvement on both quantity and frequency of drinking in college samples, that the online interventions were preferred to no intervention, and that their effects were comparable to those of alternative alcohol-related interventions.

However, for population segments other than students, the results found in literature are not yet conclusive. A recent meta-analysis focusing on online alcohol and tobacco interventions in the general population suggested positive outcomes, that is, an overall effect size (Cohen’s d) of 0.22 (95% confidence interval [CI] 0.14 - 0.29) for the alcohol interventions [12]. Close examination of the findings, however, shows that the meta-analysis contained only 3 original studies directed at decreasing alcohol use in the general adult population. All other studies were either aimed at student populations and/or at reducing tobacco use.

A number of studies did report positive effects of online self-help alcohol modules in the general adult population. A recent meta-analysis [13] reported an overall medium effect size (g = 0.40, 95% CI 0.29 - 0.50) for 9 randomized controlled trials (RCTs), including the present study, on online self-help interventions targeted at reducing alcohol intake. Most of the modules in this meta-analysis are, however, fairly time consuming, ranging from one 90-minute session [14] to a 10-week program, and some require involvement of a therapist.

The present study examines the effectiveness of Drinktest, a single 10-minute online session in which tailored feedback is delivered, with no therapist involved. (See Multimedia Appendix 1 for a screenshot of Drinktest.nl.) The aforementioned meta-analysis on online self-help reported an overall effect size of g = 0.27 (95% CI 0.11 - 0.43) if only single-session personalized feedback interventions were included in the analysis [13]. Another meta-analysis by Riper and colleagues [15] identified 14 randomized controlled trials of personalized feedback, both online and offline, aimed at reducing alcohol intake. Jointly, these had a standardized mean difference (Cohen’s d) of 0.22 (95% CI 0.16 - 0.29). These effects are appreciable, especially when taking into account that no therapist’s time is involved. Personalized feedback is assumed to be more effective than general information due to two characteristics: (1) the information is perceived as more personal and, hence, more relevant, and (2) therefore, the recipient of the information pays more attention to the key message [16].

Besides Drinktest, only one other single-session Internet-based personalized feedback intervention (Check Your Drinking or CYD) was examined in an RCT directed at the general adult population [17]. The other single-session interventions included in the meta-analysis were offered in a work setting and an emergency department of a general hospital [13]. 

Drinktest was developed by the Netherlands Institute for Health Promotion and Disease Prevention (NIGZ). Drinktest offers brief personalized feedback regarding in an individual’s personal alcohol consumption patterns. The intervention consists of various components: overview of mean weekly alcohol intake, associated health risks, self-help guidelines to reduce alcohol intake, normative feedback to compare one’s own alcohol consumption to the level of one’s own cohort. A first version of Drinktest was found to effectively reduce alcohol intake in women but not in men [18]. Since problem drinkers (16.8% men versus 4.2% women [19]) and heavy drinkers (17.3% men versus 4.1% women [20]) are predominantly men, a second version of Drinktest was developed that was tailored to males. The present study thus offers evidence from a randomized controlled trial aimed at exploring the effectiveness of the revised Drinktest in adult males. It is hypothesized that more participants in the Drinktest condition will reduce their alcohol intake relative to those in the control condition in which a general psychoeducational brochure on alcohol is offered.

## Method

### Participants

A screening questionnaire was administered to all men aged 18 to 65 (n = 9000) in two nationally representative panels consisting of 25,000 households that can receive online questionnaires. Our questionnaire contained the Quantity-Frequency Variability index of alcohol intake (QFV) [21], the Dutch version [22]. All people whose alcohol consumption exceeded the threshold specified by the Dutch guideline for low-risk drinking were invited to take part in the study [23]. People exceeding this threshold qualify as heavy drinkers [24], that is, men who consumed more than 20 units of alcohol per week (heavy drinking) and/or more than 5 units of alcohol on a single occasion on at least 1 day per week (binge drinking), where 1 unit of alcohol is equal to 10 grams of ethanol. Men were not included in the study if they had received any professional help for alcohol-related problems or any medication to reduce alcohol consumption in the 12 months preceding the study.

**Figure 1:**
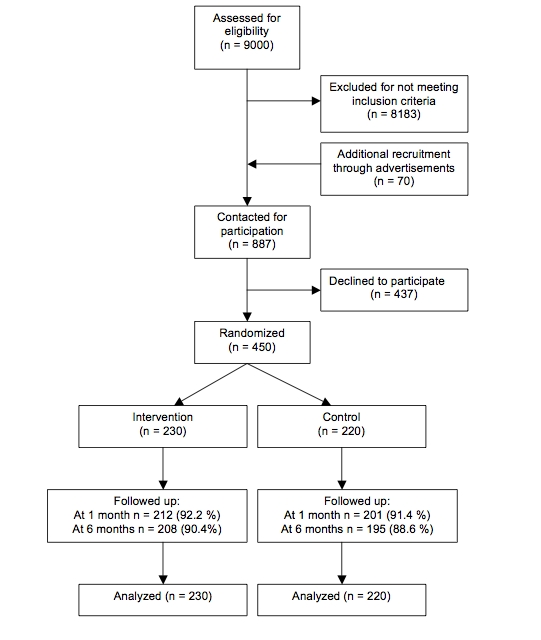
Flow chart

In total, 817 men fulfilled the inclusion criteria and were willing to consider participation in the study. Additional participants were recruited through advertisements in national newspapers, to which 70 eligible men responded. All 887 men were contacted by telephone and asked to participate. After indicating their understanding that the research included a visit to the university, a total of 450 out of the 887 (50.7%) men contacted agreed to participate and gave informed consent. After 1 month, 413 participants were successfully followed-up. Of the 37 out of the 450 (8.2%) lost to follow-up, 2 had moved away and 35 did not respond. After 6 months, 403 participants were followed up successfully. Of the 47 out of the original 450 (10.4%) lost to follow-up, 4 had moved away, 41 did not respond, and 2 had died. Figure 1 presents the flow of participants through the study.

### Procedure

At screening, participants were told a cover story to reduce the risk of response bias stemming from social desirability. Participants were told that they would judge newly developed educational materials addressing one of three possible life style topics: alcohol, smoking, or exercise. They were then told that they were randomly assigned to the alcohol group and that they were invited to evaluate the materials irrespective of their actual alcohol intake. They also received information on the procedure of the study, which consisted of one visit to the university and three written questionnaires at 0, 1, and 6 months to be filled in at home. Those responders who were eligible and willing to participate were contacted by telephone to explain to them again what participation would entail and to schedule the appointment at the behavioral laboratory. It was not revealed to the participants that their inclusion in the study was based on the degree of their alcohol intake. They were then randomized to either the computer-based personalized feedback (experimental condition) or the control group.

Prior to the appointment, participants received an informed consent form and a baseline questionnaire at their home address and were asked to bring both to their appointment. Written and signed consent was thus ensured. The baseline questionnaire included items measuring alcohol consumption and demographic characteristics.

On arrival, participants received a short standardized instruction on how to use the computer and Internet site (experimental condition) or how to read the leaflet (control condition). Participants were seated individually in a soundproof room for 20 minutes in order to ensure exposure to the educational materials and to reduce the effect of possible extra-experimental factors. Materials were offered to participants identical to real-life setting in order to maximize external validity of the study. Participants in the experimental condition completed the test online and were given the opportunity to make a printed copy of their personalized feedback. Participants in the control condition read the leaflet on paper in full color print and could take a copy home afterwards.

Next, participants received a short evaluation questionnaire with dummy questions to maintain the cover story and to determine participants’ evaluation of the educational materials. They were then given the first part of their payment (€25).

At 1 month and at 6 months after studying the educational materials, participants received postal questionnaires. The first follow-up included questions about drinking behavior as well as determinants of alcohol consumption. The second follow-up also contained measures of alcohol consumption to assess effect maintenance. All items regarding alcohol consumption included in the measurements at different time points were identical so that comparisons could be made over time. After completing and returning the last follow-up, participants received the second payment (€25).

In order to minimize dropout rates, participants were sent a first reminder to return the follow-up questionnaire after 2 weeks, were sent a second reminder with a new questionnaire after another week, and were contacted by telephone for a third reminder if needed. After the last follow-up period had ended, participants received a standard letter explaining the true objectives of the study. The study was approved by the medical ethics committee of Erasmus Medical Centre in Rotterdam (reference number MEC-2006-343).

### Intervention

Participants in the experimental condition received brief personalized feedback on alcohol use through the website www.drinktest.nl. The test is designed for adults who consume alcohol regularly or excessively and invites them to explore the possible negative consequences of their drinking behavior. The aim is 2-fold: prevention of heavy drinking and reduction of alcohol intake in heavy drinkers. In the first part of the test, respondents are asked to report their weekly alcohol consumption and number of binge drinking occasions and to indicate whether they think they consume too much alcohol and whether they intend to reduce their alcohol intake in the future. Based on this information, respondents receive the first part of the advice, which covers possible consequences of their drinking behavior. The first part of the feedback also includes a normative component in which participants can compare their alcohol consumption to that of others in the same age and gender bracket. Previous literature has revealed that including normative feedback in brief interventions aimed at reducing alcohol intake has favorable effects because people generally overestimate alcohol intake of others and underestimate their own alcohol intake [8,25]. In our study, all participants qualified as heavy drinkers; thus, all of them were invited to enter the second part of the test.

In the second part, the participants are asked questions concerning their drinking moments, drinking pattern, self-efficacy, attitude, and intention (behavioral stage according to the transtheoretical model [26]) with regard to reducing alcohol intake. Based on their answers, respondents receive personalized feedback on how to reduce alcohol intake in their specific situation. Completing the intervention takes about 10 minutes.

An early version of the intervention was designed in 2002, and its effectiveness was evaluated in a randomized controlled trial from 2005 to 2006 [18]. For the current study, a second and improved version of the website was used. Changes with regard to the former website were: (1) more tailoring to male drinking situations, since the former Drinktest was found to reduce alcohol consumption only in women [18]; and (2) the tailored advice was subdivided in smaller parts and ranked according to relevance for the participant. During the study period, March 2006 through June 2007, this new website was located in a secure area on the Internet. The first version of the website remained online, but within our study, the name of this website was never mentioned to avoid participants visiting it.

### Control Condition

Participants in the control condition were given a standard brochure (“Facts About Alcohol” [23]) developed by the Netherlands Institute for Health Promotion and Disease Prevention (NIGZ) and were asked to read it carefully. The brochure contains factual information on the biological effects of alcohol, as well as on healthy and unhealthy drinking patterns.

### Randomization

Randomization was conducted using a computer random number generator in the Statistical Package for the Social Sciences (SPSS), version 15.0. (SPSS Inc, Chicago, IL, USA). Randomization was stratified by age and educational level to ensure a good balance of these prognostically relevant characteristics of the participants across the experimental conditions. The condition to which participants were assigned was revealed to research assistants once recruitment was complete. All participants were blinded to assignment by providing them with a cover story (see “Procedure” section above).

### Outcome Measures

The primary outcome measure was heavy drinking, defined as alcohol consumption exceeding the guidelines for low-risk drinking: an average of more than 20 alcohol units per week (excessive drinking) and/or more than 5 units on a single occasion on at least 1 day per week (binge drinking). Alcohol units per day per week were assessed with the Dutch version of the QFV [21,22]. Binge drinking was measured by asking respondents how often they drink more than 5 units of alcohol on a single occasion. Answering possibilities ranged from 0, *never* to 5, *every day*. In line with the Dutch guideline, the cutoff point to assess binge drinking was set on a score of 2, indicating a frequency of at least one session of binge drinking per week.

### Power

The trial was powered to detect changes in alcohol consumption comparable to those found in a previous trial [18]. In the previous trial, a decrease in mean weekly alcohol consumption of 5.72 units (SD 15.9) was found in the experimental condition. In the control condition, participants decreased their weekly consumption with on average 1.05 units (SD 15.1). Using an alpha level of .05, 2-sided, and a power (1-beta) of .80, a total sample size of 348 men was required. To compensate for loss to follow-up, we recruited an additional 20% of the sample size. To detect changes in drinking patterns in completers-only analysis, a minimum number of 435 participants was thus needed at baseline. In our study 450 men participated.

### Analysis

To check whether randomization had resulted in two comparable groups, logistic regression analysis was used with condition as the dependent variable and a set of possible confounders (among them age and level of education) as predictors. Following the CONSORT (consolidated standards of reporting trials) statement all our analyses were conducted in agreement with the intention-to-treat (ITT) principle while imputation was used to deal with loss to follow-up. Imputation of missing values was done using the expectation-maximization algorithm of Little and Rubin [27]. This is a general method of finding the maximum-likelihood estimate of the parameters of an underlying distribution from a given data set when the data has missing values [28]. The hypothesis of the study was tested using the chi-square test. We also computed the odds ratio (OR), and the number needed to treat (NNT) as the inverse of the risk difference (RD). The OR’s were obtained under a bivariate logistic regression model of response on condition and placed in their 95% confidence intervals. The RD was obtained under a linear probability model.

Furthermore, we repeated all the analyses described above for completers only and when imputation was carried out using the last observation carried forward method. Finally, in order to assess who benefited most from the intervention at the 6-month follow-up, interaction terms were computed by calculating the products of the intervention dummy (intervention versus control) with four dichotomous variables: (1) age (18-44 vs > 44), (2) education (high vs low, ie, academic or college degree versus lower levels), (3) weekly alcohol units at baseline (< 28 versus ≥ 28), and (4) binge drinking at baseline (at least once per week versus less frequently). We then entered these interaction terms together with the corresponding main effects into the logistic regression model.

All tests were conducted at alpha = .05 (two-sided) except for the check on randomization, which was done at alpha = .10 to ensure that also relatively small baseline differences between groups in terms of age and level of education would be detected. Data were analyzed using SPSS version 15.0.

## Results

### Participants’ Characteristics

Table 1 presents participants’ characteristics and primary outcome measures at baseline. Mean age of respondents was 40.4 (SD 15.1). Overall, most men had a high level of education, but this did not differ between the two conditions. Almost half of all respondents (214/450, 47.8%) indicated they were living with a partner, and the majority of the men reported being employed (253/450, 56.5%). Mean weekly alcohol consumption at baseline was equal across both groups, with 31 units for the experimental condition and 32 units for the brochure condition. No significant differences between conditions were found on demographic characteristics and drinking patterns (see Table 1).

**Table 1:** Participant characteristics and primary outcome measures at baseline

		Experimental Condition (n = 230)	Control Condition (n = 220)	Test Result	*P* Value
**Age**				*t*_448_ = −0.17	.87
	Mean (SD)	40.6 (15.2)	40.3 (15.1)		
**Education**^a^				χ²_2_ = 1.2	.54
	Low, n (%)	36 (15.7)	43 (19.5)		
	Medium, n (%)	70 (30.4)	62 (28.2)		
	High, n (%)	124 (53.9)	115 (52.3)		
**Living arrangement**				χ²_1_ = 0.6	.44
	Living with partner, n (%)^b^	113 (49.6)	101 (45.9)		
**Employment status**				χ²_1_ = 0.02	.89
	Paid employment, n (%)^b^	128 (56.1)	125 (56.8)		
**Weekly alcohol intake in standard units**				*t*_448_ = 0.53	.60
	Mean (SD)^c^	30.9 (19.2)	31.7 (14.3)		
**Frequency of binge drinking**				*t*_44__8_ = 0.70	.49
	Mean (SD)^d^	2.1 (1.3)	2.2 (1.3)		

^a^ Low = elementary or high school, medium = occupational certificate, high = university or college degree

^b^ Includes 2 missing values

^c^ A standard unit of alcohol contains 10 grams of ethanol

^d^ Frequency of binge drinking defined as frequency of consuming more than 5 units of alcohol on at least one single occasion per week

Loss to follow-up after 1 month was 8.2% (37/450) and was evenly distributed across both conditions (n = 18 in the experimental condition and n = 19 in the control condition) (χ²_1_ = 0.98, *P* = .75). Participants (n = 8) who returned their questionnaire after 6 months but not after 1 month were regarded as completers, and after 6 months, the total loss to follow-up was 10.4% (47/450) and again equally distributed across conditions (n = 22 for the experimental condition and n = 25 for the control condition) (χ²_1_ = 0.4, *P* = .53).

Table 2 reports the participants’ characteristics at baseline for the completers and those lost to follow-up. Participants who were found to be lost to follow-up were significantly younger and more often single than completers.

**Table 2:** Comparison of the participants’ characteristics at baseline between those successfully followed up and those lost during follow-up

		Followed up (n = 403)	Lost to Follow-up (n = 47)	Test Result	*P* Value
**Age**				*t*_448_ = −3.0	.003
	Mean (SD)	41.2 (15.0)	34.2 (14.8)		
**Education**^a^, n (%)				χ²_2_ = 0.87	.65
	Low, n (%)	70 (17.4)	9 (19.1)		
	Medium, n (%)	116 (28.8)	16 (34.0)		
	High, n (%)	217 (53.8)	22 (46.8)		
**Living arrangement**				χ²_1_ = 5.29	.02
	Living with partner, n (%)^b^	199 (49.6)	15 (31.9)		
**Employment status**				χ²_1_ = 0.63	.43
	Paid employment, n (%)^b^	229 (57.1)	24 (51.1)		
**Weekly alcohol intake in standard units**				*t*_448_) = 1.7	.09
	Mean (SD)^c^	30.8 (17.0)	35.2 (17.9)		
**Frequency of binge drinking**				*t*_448_ = 1.1	.29
	Mean (SD)^d^	2.1 (1.3)	2.3 (1.2)		

^a^ Low = elementary or high school, medium = occupational certificate, high = university or college degree

^b^ Includes 2 missing values

^c^ A standard unit of alcohol contains 10 grams of ethanol

^d^ Frequency of binge drinking defined as frequency of consuming more than 5 units of alcohol on at least one single occasion per week

### Outcomes

Table 3 reports the intervention effects on the primary outcome: the percentage of men who decreased their alcohol consumption to below the Dutch guideline for low-risk drinking (to score below this guideline, a person should not exceed the limits for both weekly alcohol intake, ie, > 20 alcohol units per week, as well as for binge drinking, ie, > 5 units on a single occasion on at least one day per week). At 1 month after studying the materials, significantly more participants in the experimental condition managed to cut down on their drinking to within the guideline norms than those in the leaflet condition (97/230 or 42.2% vs 67/220 or 30.5%) (OR = 1.7, 95% CI 1.13-2.46, NNT = 8.6, χ²_1_  = 6.7, *P* = .01). These significant results were replicated under the completers-only and last observation carried forward imputation analyses (see Table 3).

**Table 3:** Change in success rates of adherence to the low-risk drinking guideline at the 1-month follow-up

		Experimental Condition		Control Condition							
		n	% Success	n	% Success	OR	95% CI	NNT^a^	χ²	*P* Value
**1-month follow-up**										
	Total sample EM^b^ imputation	230	42.2	220	30.5	1.7	1.13-2.46	8.6	χ²_1_ = 6.7	.01
	Completers-only	207	41.5	195	29.2	1.7	1.14-2.60	8.1	χ²_1_ = 6.6	.01
	Total sample LOCF^c^ imputation	230	37.4	220	25.9	1.7	1.14-2.56	8.7	χ²_1_ = 6.8	.01
**6-month follow-up**										
	Total sample EM^b^ imputation	230	45.7	220	37.3	1.4	0.97-2.06	11.9	χ²_1_ = 3.3	.07
	Completers-only	195	44.1	188	36.7	1.3	0.90-2.05	13.5	χ²_1_ = 2.2	.14
	Total sample LOCF^c^ imputation	230	37.4	220	31.4	1.3	0.88-1.93	16.7	χ²_1_ = 1.8	.18

^a^ NNT = numbers needed to treat

^b^ EM imputation = imputation based on the expectation-maximization algorithm

^c^ LOCF imputation = imputation based on last observation carried forward

At 6 months after studying the educational materials, even more participants in both conditions decreased their alcohol consumption to below the limits of heavy drinking, that is, 45.7% (105/230) in the experimental condition and 37.3% (82/220) in the control condition, but the difference between the conditions was no longer significant (χ²_1_ =3.3, *P* = .07) (Table 3). These findings were replicated under completers-only and last observation carried forward imputation analyses.

### Predictors of Favorable Outcome

Analyses of the predictor-by-treatment interaction effects showed that favorable treatment response at the 6-month follow-up was not modified by any of the patient characteristics as measured at baseline, that is, age (Wald test = 3.03, df = 1, *P* = .25, OR = 0.64, 95% CI 0.30-1.37); level of education (Wald test = 0.15, df = 1, *P* = .70, OR = 1.16, 95% CI 0.54-2.49); weekly alcohol consumption at baseline (Wald test = 0.40, df = 1, *P* = .53, OR = 0.76, 95% CI 0.33-1.76); and binge drinking at baseline (Wald test = 3.857, df = 1, *P* = .05, OR = 2.35, 95% CI 1.00-5.49).

## Discussion

### Main Findings

The results of this study show that computer-based personalized feedback is successful in decreasing the percentage of male heavy drinkers in the short run. After 1 month, participants who received the intervention were more successful than controls in bringing down their alcohol consumption, even to within the guideline norms for low-risk drinking (42% versus 31%). However, after 6 months, the success rates were 46% versus 37% for the intervention and the control condition, respectively, and did not reach statistical significance, either under an intention-to-treat analysis or the completers-only analysis.

Our findings lend partial support to the idea that computer-based personalized feedback has a more favorable effect on the reduction of heavy drinking than a standard brochure on alcohol consumption, at least in the short-term. The initial effect of the intervention is further confirmed by the number needed to be treated (NNT), estimated at 8.6, which is comparable to, for example, the NNT found in Riper and colleagues (where NNT was equal to 8.5) in a more intensive intervention directed at the same population (ie, a general population >18 years of age) [10]. Although not all the heavy drinking men reduced their alcohol intake to within the low-risk drinking guideline, a substantial number of them did. Drinking below this guideline implies lower risks for the health-related problems and excess mortality due to heavy drinking, provided that effects are maintained over time [3,29]. Treatment response at the 6-month follow-up was not predicted by age, level of education, weekly alcohol consumption at baseline, or binge drinking level at baseline. This finding indicates that Web-based personalized feedback may be well suited to a heterogeneous group of heavy drinking men, as has previously been shown by Riper and colleagues [30].

These results appear to confirm previous findings on the effectiveness of online alcohol interventions [9,12,13]. Both Cunningham’s Check Your Drinking and the Drinktest in the present study include normative feedback, a comparison of a person’s drinking to others of the same age and sex, which is considered to be one of the effective components of personalized feedback [25].

### Limitations and Strengths

The findings should be seen in the light of the limitations and strengths of our study. To begin, a substantial percentage of participants in both conditions decreased their alcohol consumption to below the Dutch guidelines for low-risk drinking. An explanation for this may be that compared with those not willing to participate in the study (dropout before randomization was 50%), participants may have been more motivated to change their alcohol intake. The fact that subjects thought they would evaluate educational materials irrespective of their actual alcohol intake, however, turns this into an unlikely explanation of the favorable effect. Also, the repeated alcohol questions participants were asked to fill out at different time points may have had an intervention-effect. Neither the possible high motivation of our subjects nor the intervention effect of the alcohol measures, however, explains the differences in alcohol consumption at follow-up between the intervention and control condition. Moreover, the effect size found in our study is similar to those reported in face-to-face inventions aimed at adults in the general population [13].

The overall loss to follow-up in our study was limited to 10%. This is a low percentage compared with the average loss to follow-up of 35% as found in meta-analyses on online alcohol interventions [11-13]. Moreover, dropout rates did not differ markedly between the intervention and control groups. We did see, however, that participants lost to follow-up were often younger and more often single. It is also possible that participants not followed up had higher alcohol consumption rates at the time of the follow-ups than those completing the study. Following the intention-to-treat principle, we handled respondents lost to follow-up in the analyses as stringently as possible by applying two different imputation techniques to estimate missing endpoints: one based on the expectation-maximization algorithm, the other conservatively based on last observation carried forward. In addition, we conducted completers-only analysis. Each time, we were able to replicate research findings. This attests to the robustness of our findings.

A limitation of this study is that we relied on self-reported measures. However, Del Boca and Darkes [31], in their review of the validity of self-reports of alcohol consumption concluded, “self-report measures have demonstrated reasonable levels of reliability and validity.” Also, we do not expect that any bias that may stem from self-report would be different in the experimental condition as compared with the control condition.

Due to the nature of the educational materials in our study, blinding of participants was not possible. This may have led respondents to underreport their alcohol consumption at follow-up. We tried to minimize the possibility of social desirability bias in the primary outcome by using a cover story. Respondents were not informed of the true objectives of the study until after the last follow-up questionnaires were received.

An additional drawback of the present study may be that the sample was limited to adult men, and the results may, therefore, not simply be generalized to women. However, restricting the present study to men was a deliberate choice, since in our previous study with an earlier version of the intervention we demonstrated that it was beneficial to women but not to men [18]. Moreover, heavy drinking is more prevalent in men [19,20].

Finally, it may be seen as a limitation of our study that we invited participants to visit our laboratory, which may threaten the external validity. This may, for example, have induced participants to spend all the available time actually reading the tailored advice, whereas if they were to conduct the intervention in their home environment, participants might not complete the entire intervention due to possible distractions or time constraints. Similarly, in a private setting, participants may not be able to ask for help, whereas in the laboratory situation a research assistant was present if they needed any support on the use of the computer and the Drinktest website. However, at this stage it was also a conscious choice to conduct a randomized controlled trial under laboratory conditions in order to assess the efficacy of the intervention after which an effectiveness study with a pragmatic randomized trial could be conducted. Furthermore, a recent meta-analysis [13] did not find any significant differences when comparing the effects of interventions based on where they were conducted (ie, research or health centre vs participants’ homes). Nevertheless, it cannot be ruled out that the laboratory setting may have helped to increase the effect of both the Drinktest and the brochure, but is unlikely to have induced a differential effect in this randomized trial favoring one condition over the other.

### Conclusions

Personalized online feedback on alcohol appears to be an effective and fairly easy way to change unhealthy drinking patterns in adult men, at least in the short-term. Drinktest.nl yearly draws about 90,000 male visitors. Of these, 70% (63,000) report to be heavy drinkers, and 40% (25,200) of these heavy drinkers complete the test, implying that they actually receive the complete tailored feedback. Based on the NNT at the 6-month follow-up, assuming that the revised Drinktest will attract the same number and type of visitors each year and assuming that conducting Drinktest in a private setting would generate the same effects, this would imply that more than 2000 men per year (n = 2117) will successfully reduce their alcohol intake during at least 6 months as a consequence of spending 10 minutes of their time on Drinktest.nl. Offering personalized feedback on alcohol through highly accessible Internet sites may thus contribute to generating health gains at the population level in an efficient and economically affordable way. In fact, Smit and colleagues calculated that introducing evidence-based eHealth interventions such as Drinktest into the Dutch health care system would substantially improve the cost-effectiveness of the system for alcohol use disorders overall [32].

## Multimedia Appendix 1

Screenshot of Drinktest.nl

## Abbreviations

CI: confidence interval
CONSORT: consolidated standards of reporting trials
CYD: Check Your Drinking
ITT: intention-to-treat
NIGZ: Netherlands Institute for Health Promotion and Disease Prevention
NNT: number needed to treat
OR: odds ratio
QFV: Quantity-Frequency Variability (index of alcohol intake)
RD: risk difference
RCT: randomized controlled trial

## References

[1] World Health Organization (2007). WHO Expert Committee on Problems Related to Alcohol Consumption, Second Report.

[2] World Health Organization (2002). The World Health Report 2002: Reducing Risks, Promoting Healthy Life.

[3] World Health Organization (2004). Global Status Report on Alcohol 2004.

[4] Holman CD, English DR, Milne E, Winter MG (1996). Meta-analysis of alcohol and all-cause mortality: a validation of NHMRC recommendations. Med J Aust.

[5] Smit F, Cuijpers P, Oostenbrink J, Batelaan N, de Graaf R, Beekman A (2006). Costs of nine common mental disorders: implications for curative and preventive psychiatry. J Ment Health Policy Econ.

[6] Moyer A, Finney JW (2004). Brief interventions for alcohol problems: factors that facilitate implementation. Alcohol Res Health.

[7] Andrews G, Issakidis C, Sanderson K, Corry J, Lapsley H (2004). Utilising survey data to inform public policy: comparison of the cost-effectiveness of treatment of ten mental disorders. Br J Psychiatry.

[8] Bewick BM, Trusler K, Barkham M, Hill AJ, Cahill J, Mulhern B (2008). The effectiveness of web-based interventions designed to decrease alcohol consumption--a systematic review. Prev Med.

[9] Vernon ML (2010). A review of computer-based alcohol problem services designed for the general public. J Subst Abuse Treat.

[10] Riper H, Kramer J, Smit F, Conijn B, Schippers G, Cuijpers P (2008). Web-based self-help for problem drinkers: a pragmatic randomized trial. Addiction.

[11] Carey KB, Scott-Sheldon LA, Elliott JC, Bolles JR, Carey MP (2009). Computer-delivered interventions to reduce college student drinking: a meta-analysis. Addiction.

[12] Rooke S, Thorsteinsson E, Karpin A, Copeland J, Allsop D (2010). Computer-delivered interventions for alcohol and tobacco use: a meta-analysis. Addiction.

[13] Riper H, Spek V, Boon B, Conijn B, Kramer J, Martin-Abello K, Smit F (2011). Effectiveness of E-Self-help Interventions for Curbing Adult Problem Drinking: A Meta-analysis. J Med Internet Res.

[14] Hester RK, Squires DD, Delaney HD (2005). The Drinker's Check-up: 12-month outcomes of a controlled clinical trial of a stand-alone software program for problem drinkers. J Subst Abuse Treat.

[15] Riper H, van Straten A, Keuken M, Smit F, Schippers G, Cuijpers P (2009). Curbing problem drinking with personalized-feedback interventions: a meta-analysis. Am J Prev Med.

[16] Kreuter MW, Oswald DL, Bull FC, Clark EM (2000). Are tailored health education materials always more effective than non-tailored materials?. Health Educ Res.

[17] Cunningham JA, Wild TC, Cordingley J, van Mierlo T, Humphreys K (2009). A randomized controlled trial of an internet-based intervention for alcohol abusers. Addiction.

[18] Boon B, Huiberts A, Meijer SA, Smit F, Schoemaker CG, Cuijpers P (2006). Preventie problematisch alcoholgebruik. Gezond verstand. Evidence-based preventie van psychische stoornissen [Common sense. Evidence-based prevention of mental disorders] Volksgezondheid Toekomstverkenning (VTV) Themarapport.

[19] Van Dijck D, Knibbe RA (2005). De Prevalentie van Probleemdrinken in Nederland. Een Algemeen Bevolkingsonderzoek.

[20] Statline Gezondheid, leefstijl, gebruik van zorg.

[21] Cahalan D, Cisin IH, Crossley HM (1969). American Drinking Practices: A National Study of Drinking Behavior and Attitudes.

[22] Lemmens P, Tan ES, Knibbe RA (1992). Measuring quantity and frequency of drinking in a general population survey: a comparison of five indices. J Stud Alcohol.

[23] Van Bergen S, van Boxtel T, Canter C (2004). Feiten over alcohol Facts about alcohol.

[24] World Health Organization Lexicon of alcohol and drug terms published by the World Health Organization.

[25] Chan KK, Neighbors C, Gilson M, Larimer ME, Alan Marlatt G (2007). Epidemiological trends in drinking by age and gender: providing normative feedback to adults. Addict Behav.

[26] Prochaska JO, DiClemente CC (1992). Stages of change in the modification of problem behaviors. Prog Behav Modif.

[27] Little RJA, Rubin DB (1987). Statistical Analysis with Missing Data.

[28] Tabachnick BG, Fidell LS (2007). Using Multivariate Statistics. 5th edition.

[29] Anderson P, Baumberg B (2006). Alcohol in Europe: A Public Health Perspective.

[30] Riper H, Kramer J, Keuken M, Smit F, Schippers G, Cuijpers P (2008). Predicting successful treatment outcome of web-based self-help for problem drinkers: secondary analysis from a randomized controlled trial. J Med Internet Res.

[31] Del Boca FK, Darkes J (2003). The validity of self-reports of alcohol consumption: state of the science and challenges for research. Addiction.

[32] Smit F, Lokkerbol J, Riper H, Majo C, Boon B, Blankers M, Poznyak V (2011). Modelling the cost-effectiveness of health care systems for alcohol use disorders. J Med Internet Res.

