# Supplementary material for: Curbing Alcohol Use in Male Adults Through Computer Generated Personalized Advice: Randomized Controlled Trial
Source: J Med Internet Res. 2011 Jun 30;13(2):e43. doi: 10.2196/jmir.1695 (PMC3221373; doi:10.2196/jmir.1695)
Supplement: Supplementary file 1 [file jmir_v13i2e43_app1.pdf]

Trimbos instituut

ADVIES OP MAAT

www.drinktest.nl

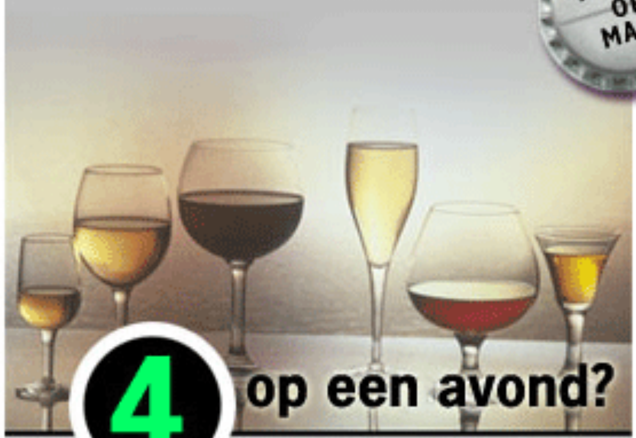

OVER DEZE TEST / TRIMBOS-INSTITUUT

(Deze test vereist dat uw browser cookies accepteert)

Drinkt u regelmatig alcohol en wilt u weten of uw drinkgedrag een risico is voor uw gezondheid? Neem dan tien minuten tijd voor deze test.

De eerste vragen gaan vooral over de hoeveelheid alcohol die u drinkt en geven u hierover advies. Afhankelijk van de uitslag kunt u vervolgens nog één of meerdere delen van de test maken en ontvangt u meer advies en tips.

Wat is uw favoriete alcoholhoudende drank?

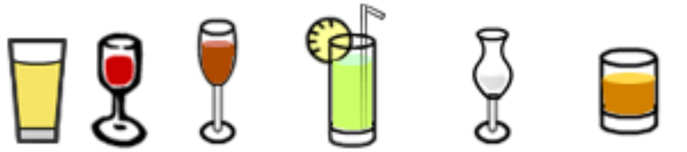

Bier | Wijn | Sherry-Port | Cocktail-Mixdrank | Jenever | Whiskey

START
